# From Familiar Faces to New Spaces: Perceived Social Network Change and Its Association With Loneliness During the Transition to University

**DOI:** 10.1002/jad.70249

**Published:** 2026-08-06

**Authors:** Hannah Rachael Slack, Nicola Byrom, Chloe Bate, Aleksandra Hościłowicz, Sophie Homer, Delia Fuhrmann, Juliet Foster, Sarah Crook, Emily Long

**Affiliations:** ^1^ Department of Psychology Institute of Psychiatry, Psychology and Neurosciences, King's College London London UK; ^2^ MRC/CSO Social and Public Health Sciences Unit, University of Glasgow Glasgow UK; ^3^ School of Psychology, University of Plymouth Plymouth UK; ^4^ Department of Psychology Institute of Psychiatry, Psychology and Neuroscience, King's College London London UK; ^5^ Department of History Heritage and Classics, Swansea University Swansea UK

**Keywords:** higher education, loneliness, social network, students, university

## Abstract

**Background:**

Loneliness among young people is a growing concern, and life transitions can create significant upheaval in social networks. The scale of these changes and their association with loneliness remains underexplored.

**Objective:**

This study examined retrospectively recalled changes in network composition and structure across the transition to university and explored associations with loneliness.

**Methods:**

We used social network interviews to assess 79 first‐year students' networks before and after starting university, measuring loneliness post‐transition; 77% of the sample were 18–21 years and 72% were female.

**Results:**

Participants recalled substantial change in their network (network churn); on average 58% of social connections were gained or lost. Network change was normal and widespread. Networks became larger, less dense, more geographically dispersed, less emotionally close, more heterogeneous in composition, while perceived support remained broadly stable at population level. Greater loneliness was associated with perceived reductions in network support (*B* = −1.03, SE = 0.48) and greater ethnic heterogeneity within social networks (*B* = 1.26, SE = 0.41).

**Conclusion:**

This study highlights the substantial social restructuring that accompanies the transition to university. Young people reported marked changes in their social networks, characterized by expansion, diversification, and considerable network churn, suggesting that university entry represents a distinctive developmental transition requiring adaptation to new social environments.

## 1 Introduction

The transition from late adolescence to emerging adulthood involves profound changes in social relationships. As young people leave school, relocate, and enter new social environments, their personal networks often undergo substantial restructuring. Loneliness is a growing concern for emerging adults (ages 18–25) and transitions are a point of risk (Matthews et al. 2018).

As concerns grow around loneliness, we need new ways of understanding the issue. Loneliness is commonly defined as the emotional state experienced because of a perceived discrepancy between an individual's desired and actual social relationships, in terms of both relational quantity and quality (Perlman and Peplau 1981; Qualter et al. 2015). To understand loneliness through this social lens we need to consider how social landscapes shift during transitions. Identifying social predictors of loneliness may inform interventions at both structural and individual levels.

Starting university is one socially disruptive transition. Starting university often involves moving away from home, leaving familiar environments, living independently, and losing daily contact with close friends and family (Juvonen 2014). This leads to a significant reshaping of social networks (Baggio et al. 2016; Thomas et al. 2020), a process referred to as network churn; connections are lost and new ones formed (Weiss et al. 2022). Some degree of network churn is both normal and healthy during life transitions, as individuals replace old connections with new ones that serve similar social functions. However, excessive churn is expected to leave students vulnerable to loneliness and social isolation (Fischer and Offer 2020; Kyeremeh and Schafer 2024).

Two different hypotheses can be generated for how social networks may change over the transition to university. Social Convoy Theory suggests that networks remain relatively stable (Antonucci et al. 2014; Wrzus et al. 2013). Conversely, qualitative research suggests transition induced network reconfiguration, where network change may be more radical (Priestley et al. 2025). We consider these two possibilities in turn.

Social Convoy theory suggests that while young people's social networks undergo substantial shifts during the transition to adulthood, the overall size and composition of the network will remain stable (Weiss et al. 2022). As people drift apart or their needs change, relationships may be dropped (Neves et al. 2019). However, social connections providing practical assistance, founded on a high‐level of trust, or embedded within stable social contexts are most likely to survive life transitions (Kyeremeh and Schafer 2024). As young people lose connections, they often replace these with new ones that fulfill similar needs, leading to *overall* network stability. This aligns with social convoy theory, which conceptualizes people's social networks as “convoys” of significant others who accompany them throughout life, providing emotional, practical, or informational support (Antonucci et al. 2014; Wrzus et al. 2013). Importantly, while the passengers in the convoy—that is, members of the network—may change over time, the convoy's shape and structure is stable (Antonucci et al. 2014).

In contrast to the network stability predicted by social convoy theory, transition induced network reconfiguration account proposes that major changes in role and social context can temporarily alter the opportunities and constraints governing relational formation, allowing more radical changes in young people's social networks. Leaving behind the structured environment of school and home, emerging adults often encounter greater freedom and opportunity to choose their friends (Priestley et al. 2025). This newfound autonomy may lead students to shed dissimilar connections and create a more homogenous network, with preferences for similarity in age and ethnicity (Jeroense et al. 2024).

How will changes in social network relate to social connection and loneliness? Through the transition to university, consistent supportive relationships serve as a vital buffer against a range of academic, social, and financial stressors (Byrom et al. 2025; Fischer and Offer 2020). Different relationships serve distinct purposes, with family relationships offering stability and emotional grounding, while new peer relationships provide contextually relevant support for academic and social integration (Weiss et al. 2022). Forming new friendships and support networks can foster belonging, ease adjustment, and reduce the risk of loneliness (Maunder 2018). Yet building new relationships takes time, effort, and opportunity—resources that are not equally available (Baggio et al. 2016; Priestley et al. 2025; Thomas et al. 2020). With autonomy to choose your network, people tend to form relationships with those with shared characteristics and backgrounds, a tendency referred to as homophily. This can foster a shared sense of belonging (Jeroense et al. 2024). However, in institutions that have historically catered to affluent groups, students who do not see themselves represented within the majority demographic may struggle to experience belonging. Misalignment with the institutional norm can foster ‘institutional loneliness’ and hinders the formation of meaningful connections (Crook 2025).

In this study we examine the extent to which young adults perceive their social network to change when moving to university, testing the idea that these changes involve high levels of network churn. Next, we test the hypothesis that perceived network churn is associated with loneliness in the student population. We then compare how the retrospectively recalled pre‐university and current university networks differ in terms of their size and composition. Finally, we fit a series of exploratory models to identify associations with loneliness during this critical transition, testing the hypotheses that loneliness will be associated with (1) decreased network support and (2) decreased homophily, operationalised as increased gender or ethnicity heterogeneity.

## 2 Methods

### 2.1 Design

This was a non‐experimental observational study with descriptive and correlational aims, using a retrospective within‐person egocentric social network design. The egocentric network captures an individual's social contacts and the relationships between those contacts (Crossley et al. 2015). Participants described both their pre‐university and current university networks during a single post‐transition interview, allowing comparison of retrospectively recalled network characteristics across the transition. The study was therefore retrospective rather than prospectively longitudinal. The study was approved by the King's College London, PNM Research Ethics Panel (Reference: LRS/DP‐22/23‐39056) and University of Glasgow, College of Medical, Veterinary & Life Sciences Ethics Committee (Reference: 200230106).

### 2.2 Participants

In total, 90 first year or foundation year undergraduate students, from universities across the UK, completed a social network interview alongside two surveys. Table 1 shows sample demographics. Participants were recruited through university newsletters and social media, inviting students to take part in a study about their social relationships. Participants attended 24 different universities, including both Russell group and post‐92 institutions. Informed consent was collected prior to completion of the first survey. At the second survey, participants opted into being contacted for a social network interview, providing informed consent for the interview.

**Table 1:** A summary of sample demographics.

Demographic characteristic	Full sample *N*	Analytic sample *N*
Gender		
Woman	65	57
Man	17	17
Non‐Binary	7	5
Age		
≤ 21	69	61
≥ 22	9	7
Age missing data	12	11
Ethnicity		
White	43	37
Asian	26	22
Black	8	8
Mixed/Other/PNTS	13	12
Student status		
Domestic	45	41
International	23	18
PNTS	22	20
Total	90	79

*Note:* PNTS refers to “Prefer not to say.” For ethnicity, “Other” comprised of Arab, Latinx, and Assyrian.

### 2.3 Procedure

The timeline for data collection is summarized in Figure 1. Egocentric social network data was collected in three steps—name generation, name interpretation and alter‐alter matrix—within a Social Network Interview via Zoom. Interviews were conducted by a trained, experienced fieldworker, supported by Network Canvas, an open‐source software for surveying networks (Hogan et al. 2016). In the name generator phase of the interview participants were asked to name: (a) people they had interacted with in the last 2 weeks, (b) people they considered themselves to be connected to but had not interacted with recently, and (c) people they had frequently interacted with but did not necessarily know well. There was no limit on the number of people participants could name (Maya Jariego 2018). In the name interpreter phase, participants then indicated, for each named person, whether the person was part of their social network in the Spring or Summer before starting university (their pre‐university network) and/or in their current network while studying at university (their university network). Participants then answered follow‐up questions about each contact (see Table 2). Finally, in the alter‐alter matrix phase, participants mapped whether each member of their network knew the other members of the network. Demographic information was collected early in the academic year (Time 1) via an online survey hosted on Qualtrics. Loneliness was measured later in the year (Time 2). The Social Network Interview was scheduled a few weeks after the loneliness survey.

**Figure 1:**
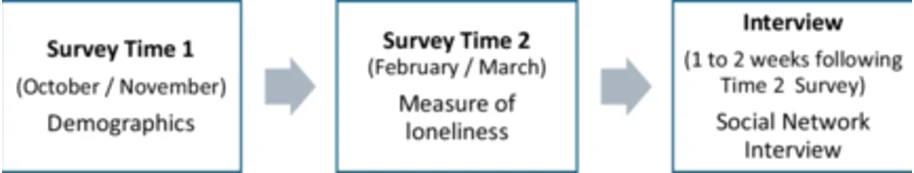
Timeline for data collection.

**Table 2:** A summary of the constructs measured.

Construct	Approach taken
**1. Individual‐level characteristics**	
Demographics	Gender (Woman, Man, Non‐binary, Unsure/PNTS) and ethnicity (White, Asian, Black, Mixed, Other, PNTS)
Loneliness	UCLA‐3 (Russell 1996), a three item, 5‐point Likert scale (Never—Very often; (Qualter et al. 2021). Items were summed to produce a total score ranging from 3 to 15. A higher score indicates greater loneliness. Robust internal reliability in this sample: *α* = 0.88
**2. Social contact‐level attributes**	
Homophily, operationalised as decreased heterogeneity of contacts	External–Internal (E‐I) indexes were calculated for each participant separately for **gender** and **ethnicity**, reflecting the extent to which an individual's social contacts share the same demographic characteristic as them. Scores range from −1 (all contacts match) to +1 (all contacts differ); (Krackhardt and Stern 1988)
Diversity of contacts	Shannon's entropy scores were calculated for **gender** and **relationship type** (i.e., friend, family, partner, and acquaintance) to capture the diversity of social contacts along each dimension. Higher scores represent greater diversity (Shannon 1948). Minimum score = 0, all contacts fall into a single category, there is no diversity. Maximum score for gender (4 categories) ≈ 1.10 and for relationship type (4 categories) ≈ 1.39. Maximum score occurs when all categories are equally common
**3. Relationship features**	
Perceived geographical distance	For each social contact, perceived geographical distance was rated from 1 (*We live together*) to 6 (*They don't live in the UK*). Ratings were averaged across all contacts in the relevant network
Perceived support	For each social contact, participants rated the emotional or social support provided by that person, from 1 (*They don't support me*) to 3 (*They support me a lot*). Ratings were averaged across all contacts in the relevant network
Perceived closeness	For each social contact, perceived closeness was rated from 1 (*Not very close*) to 5 (*Very close*). Ratings were averaged across all contacts in the relevant network
Relative offline/online interaction frequency	Frequency of offline interactions and frequency of online interactions were measured, with a single item, on a 0 (*Never*) to 5 (*Most days of the week*) scale. These were averaged across all contacts in the relevant network for each participant. Relative score=average offline interaction frequency−average online interaction frequency. Greater difference scores indicate more offline interaction than online interaction
Percentage online‐only contacts	The number of contacts with whom the participant reported some online interaction but no offline interaction, divided by the total number of contacts in the relevant network and multiplied by 100
**4. Network‐level measures**	
Network size	Total number of contacts mentioned by the participant
Network density	The proportion of connections (ties) in a network relative to the possible connections if everyone were connected to everyone else. Higher scores indicate a more interconnected network
Network churn	Measure of network stability reflecting the extent to which connections lost in the transition to university were replaced (Weiss et al. 2022). $\text{Network churn}\;=\;(C_{\text{pre}}+C_{\text{current}})/C_{\text{total}}$, where *C*_pre_ is the number of contacts present only in the pre‐university network, *C*_current_ is the number of contacts present only in the current network and *C*_total_ is the number of contacts across both networks. Churn scores range from 0 (no change) to 1 (complete change)

## 3 Materials and Measures

We captured four domains of social relationships pertinent to students' feelings of loneliness (Vacca 2020): (1) individual characteristics, such as gender and ethnicity; (2) attributes of social contacts, including perceived gender and ethnicity; (3) relationship features, such as relationship type (e.g., family vs. peer) and perceived closeness; and (4) network‐level measures, such as density (i.e., how tightly knit the group is). A comprehensive list of variables is provided in Table 2. Of the variables included in the analyses, missingness ranged from 0% to 5.56% (see Appendix A). Attributes of the participants' social contacts were derived from their perceptions rather than objective measures (e.g., the participant may not know the precise age, gender, or ethnicity of a social contact). This perception of the network is of intrinsic interest, as individuals' understanding of their social network can influence their behavior (Kenney et al. 2017).

### 3.1 Analyses

Except for demographics and loneliness, measures were derived from the egocentric network data at the participant‐network level. For each participant, compositional measures, relationship features, network size and network density were calculated separately for the retrospectively reported pre‐university and current university networks; network churn was calculated across the two networks. Ratings of geographical distance, support, closeness, and offline and online interaction frequency were collected for each social contact and averaged across all contacts belonging to the relevant network. These scores therefore describe the characteristics of the average network member, including acquaintances and weaker ties, rather than indicating whether the participant had any close or supportive relationships. E‐I indices and Shannon entropy scores summarized network composition, while network size, density and churn captured network structure and turnover, as defined in Table 2. For analyses of perceived change, pre‐university values were subtracted from university values, such that positive scores represented an increase following the transition.

All analyses were conducted in RStudio version 4.3.0. We used bivariate correlations, paired samples *t*‐tests, and multiple linear regressions. Normality was assessed using Shapiro‐Wilks tests, Q‐Q plots and histograms. Non‐parametric tests were used when the data violated the normality assumption. For linear regressions, all continuous predictors were mean‐centered and scaled (Gelman and Hill 2008), and categorical variables (gender, ethnicity and student status) were Helmert coded to allow for stepwise comparisons between factor levels (Wendorf 2004). The regression residuals were normally distributed in all models. Missing data was handled using listwise deletion, resulting in a complete analytical sample across all analyses; the analytic sample, as compared with the full sample, is shown in Table 1.

Exploratory multiple linear regression was conducted to model loneliness at university, with key attributes of social relationships as predictors. Power analysis indicated that the regression model was powered at 90% to detect a large effect for the combined set of predictors, assuming *α* = 0.05. Model 1 used the participant's pre‐university network. Model 2 used the participant's university network. Model 3 drew on change metrics. All models were run with (b) and without (a) controlling for demographic factors. Both sets of models were conducted as social network characteristics are often socially patterned and correlated with demographic and contextual factors; associations may therefore be sensitive to model specification and adjustment for covariates. VIF > 5 for Gender Heterogeneity and Diversity in Model 2, indicating potential multicollinearity (see Appendix A2), thus these variables were removed from analysis sequentially (giving Model 2_a_^1^ and Model 2_a_^2^).

## 4 Results

Participants in the analytical sample provided data on 1381 social contacts, with average of 17 social contacts each (SD = 9.21, *range* = 6–60).

### 4.1 Network Churn

On average, participants recalled 58% of social connections as either dropped from the network or newly formed across the transition. This pattern was consistent across demographic groups, though Black students experienced lower churn (see Table 3). Students' experience of network churn was not significantly related to their feelings of loneliness, *r*_*s*_(79) = −0.11, *p* = 0.336 (see Figure 2).

**Table 3:** Descriptive summary of network churn across the sample and within student subgroups.

Demographic characteristic	*M (SD)*	*Min*	*Max*
Gender			
Woman	0.58 (0.17)	0.16	0.88
Man	0.62 (0.19)	0.11	0.92
Non‐Binary	0.53 (0.32)	0.00	0.79
Ethnicity			
White	0.58 (0.17)	0.11	0.92
Asian	0.58 (0.19)	0.25	0.86
Black	0.47 (0.27)	0.00	0.82
Mixed	0.68 (0.12)	0.56	0.82
Other	0.65 (0.17)	0.42	0.78
PNTS	0.69 (0.27)	0.50	0.88
Student status			
Domestic	0.54 (0.19)	0.11	0.86
International	0.66 (0.10)	0.42	0.88
PNTS	0.59 (0.21)	0.00	0.92
Analytical sample	0.58 (0.19)	0.00	0.92

**Figure 2:**
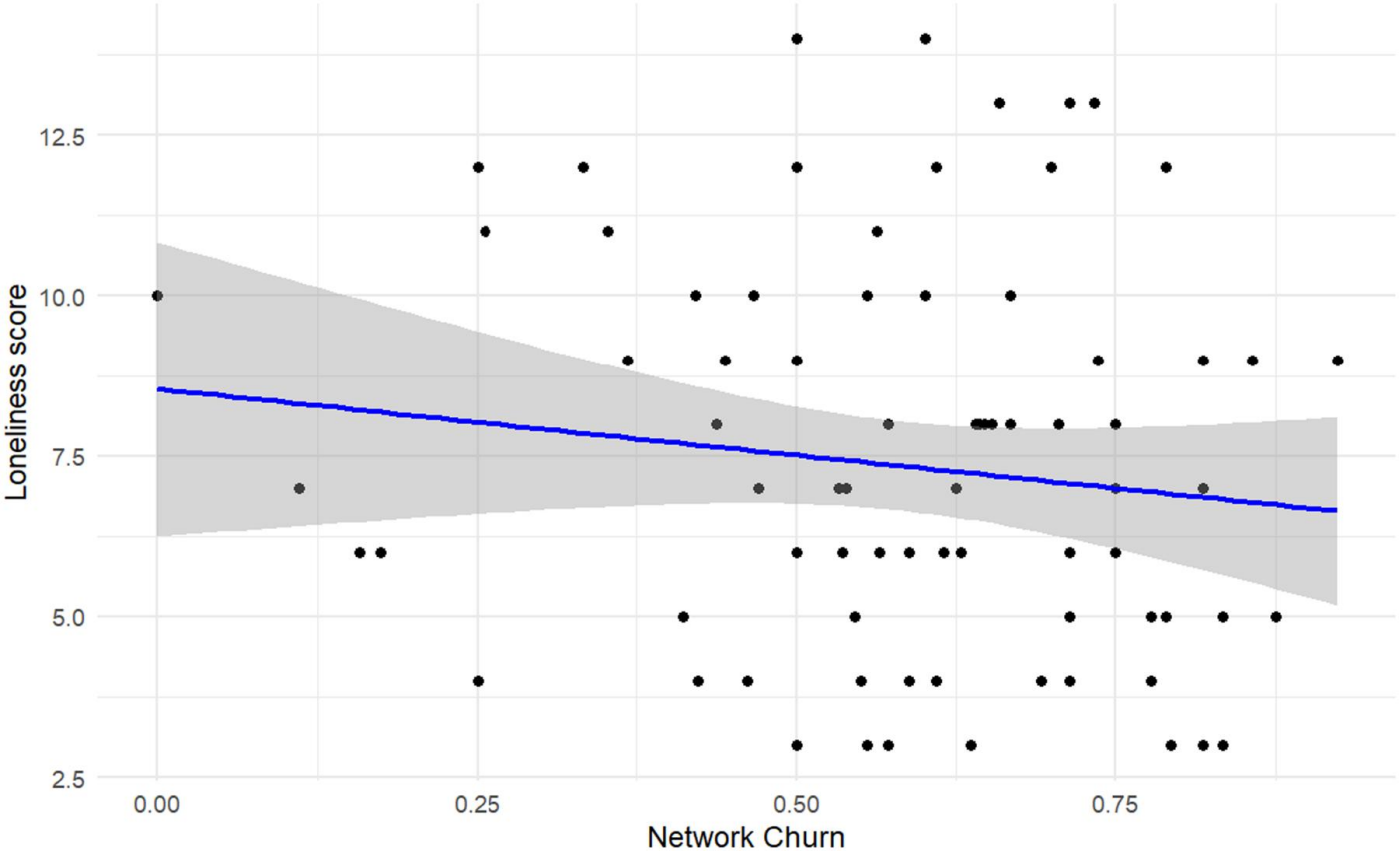
Relationship between Network Churn and Loneliness Scores.

### 4.2 Comparison Between Networks

As summarized in Table 4, participants perceived their social networks to change through the transition to university. Networks became larger (network size) and less dense. Ethnic homophily decreased; university networks included more contacts from ethnic backgrounds different to the participant's own, reflected as increased ethnicity EI index. Relationship type (e.g., friendships, familial connections) diversity also increased (relationship type entropy), indicating a broader spread of relationship categories. Change in gender, ethnicity and relationship type diversity is shown in Figure 3. While networks became more gender diverse, as shown in Figure 3, this is primarily due to small increase in participant uncertainty about the gender of new contacts in their university network. Students reported that they lived further away from (average geographical distance) and felt less close to (average closeness) their contacts than they did before university. Importantly, average support from connections remained stable.

**Table 4:** Results of paired samples *t*‐test and Wilcoxon signed rank tests comparing pre‐university and current networks.

Parameter	Pre‐university network M (SD)	University network M (SD)	Test statistic$	Effect size [95% CIs]
Loneliness	—	7.34 (3.10)	—	—
Ethnicity heterogeneity (EI index)	−0.49 (0.55)	−0.28 (0.53)	−4.94***	−0.56 [−0.79, −0.32]
Gender heterogeneity (EI index)	−0.36 (0.44)	−0.32 (0.41)	922	−0.26 [−0.48, 0.00]
Relationship type diversity (entropy)	0.82 (0.40)	0.93 (0.39)	−5.84***	−0.66 [−0.90, −0.41]
Gender diversity (entropy)	0.82 (0.40)	0.93 (0.39)	615.50**	−0.50 [−0.68, −0.28]
Average geographical distance	2.91 (0.86)	4.13 (0.65)	198***	−0.86 [−0.91, −0.77]
Average closeness	3.79 (0.55)	3.48 (0.56)	2501.50***	0.62 [0.44, 0.76]
Average support	1.31 (0.40)	1.30 (0.31)	1560.50	0.12 [−0.13, 0.36]
Relative offline/online interaction frequency	−0.01 (1.01)	−0.40 (1.00)	2.89	0.33 [0.10 0.55]
Percentage of online only relationships	5.24 (9.83)	7.34 (12.76)	263.50	−0.21 [−0.58, 0.24]
Network size	9.84 (5.33)	14.91 (8.82)	78***	−0.94 [−0.97, −0.91]
Network density	0.52 (0.24)	0.30 (0.14)	2926***	0.90 [0.84, 0.94]

*Note:* *p < 0.05; **p < 0.01; ***p < 0.001. All *p*‐values were Bonferroni corrected for multiple comparison. Test Statistic refers to a *t*‐test (t) or Wilcoxon (W) statistic. Ethnicity heterogeneity, relationship type diversity, and relative offline/online interaction frequency were assessed using paired samples *t*‐tests; effect sizes refer to Cohen's *d*. For paired *t*‐tests, the sign of *t* reflects the direction of the comparison (pre‐university minus university): negative values indicate that the university mean exceeded the pre‐university mean, whereas positive values indicate the opposite. All other parameters were assessed using Wilcoxon signed‐rank tests; effect sizes refer to the rank‐biserial correlation coefficient (*r*), with a range of –1 to 1.

^$^ Analyses included both paired samples *t*‐tests and Wilcoxon signed‐rank tests.

**Figure 3:**
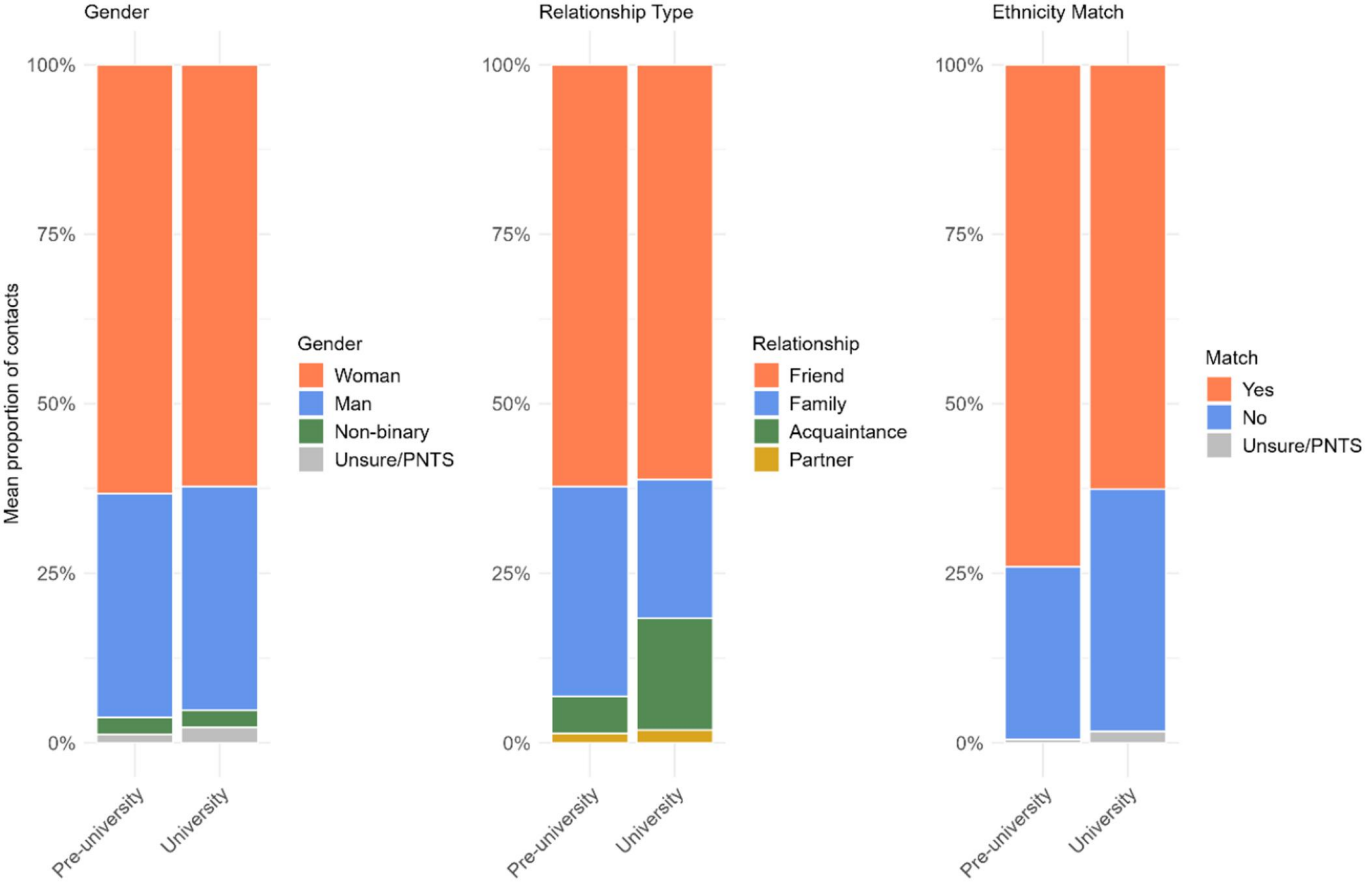
Change in network composition from pre‐university to university, showing the distribution of contacts by gender, relationship type, and ethnicity match with the participant.

### 4.3 Network Characteristics Associated With Loneliness

Multiple linear regression analyses are reported in Tables 5, 6, and 7. Pre‐university network characteristics (Model 1; Table 5) accounted for 13% of variance in loneliness, *F*(11, 67) = 0.89, *p* = 0.557. The current university network (Model 2; Table 6) accounted for 14% of the variance in loneliness, *F*(10, 68) = 1.06, *p* = 0.403. Notably, neither of these models were statistically significant, indicating that caution is needed in interpretation.

**Table 5:** Findings from regression models assessing the association between pre‐university network characteristics (Model 1) and loneliness, with (b) and without (a) controlling for demographics.

Parameter		Model 1a	Model 1b
Gender heterogeneity (EI index)		1.20 (0.58)*	0.89 (0.66)
Ethnicity heterogeneity (EI index)		−0.35 (0.39)	−0.59 (0.48)
Gender diversity (entropy)		−1.20 (0.59)*	−1.29 (0.65)
Relationship type diversity (entropy)		0.66 (0.49)	0.48 (0.55)
Average geographical distance		0.10 (0.38)	−0.08 (0.43)
Average support		0.30 (0.46)	0.24 (0.55)
Average closeness		−0.34 (0.48)	−0.54 (0.51)
% Online only relationships		−0.18 (0.42)	−0.30 (0.44)
Relative offline/online interaction frequency		0.15 (0.39)	0.24 (0.43)
Network density		−0.23 (0.44)	−0.42 (0.47)
Network size		0.10 (0.48)	0.16 (0.50)
Gender (ref: Woman)	Man		0.19 (0.51)
	Non‐Binary		0.86 (0.66)
Ethnicity (ref: White)	Asian		0.30 (0.56)
	Black		−0.21 (0.45)
	Mixed		0.84 (0.42)
	Other		−0.01 (0.34)
	PNTS		−0.33 (0.46)
Student status (ref: Domestic)	International		0.55 (0.53)
	PNTS		0.03 (0.32)
*R* ^*2*^ *(residual SE)/adjusted R* ^*2*^		0.13 (3.13)/−0.02	0.22 (3.17)/−0.05
*F(df)*		0.89 (11,67)	0.83 (20,58)

*Note:* *p < 0.05. Showing coefficient (SE).

**Table 6:** Findings from regression models assessing the association between university network characteristics (Model 2) and loneliness, with (b) and without (a) controlling for demographics.

Parameter		Model 2a^a^	Model 2a^b^	Model 2b^a^	Model 2b^b^
Gender heterogeneity (EI index)		—	0.27 (0.38)	—	−0.49 (0.60)
Ethnicity heterogeneity (EI index)		0.35 (0.41)	0.37 (0.41)	0.34 (0.54)	0.30 (0.55)
Gender diversity (entropy)		−0.10 (0.40)	—	−0.73 (0.52)	—
Relationship type diversity (entropy)		−0.03 (0.45)	−0.15 (0.43)	0.43 (0.51)	0.28 (0.51)
Average geographical distance		0.17 (0.40)	0.12 (0.40)	0.49 (0.46)	0.58 (0.47)
Average support		−0.55 (0.43)	−0.55 (0.43)	−0.63 (0.46)	−0.66 (0.47)
Average closeness		−0.33 (0.46)	−0.36 (0.46)	−0.29 (0.51)	−0.24 (0.52)
% Online only relationships		−0.06 (0.42)	−0.13 (0.42)	−0.43 (0.47)	−0.40 (0.48)
Relative offline/online interaction frequency		0.45 (0.45)	0.34 (0.46)	0.53 (0.50)	0.54 (0.51)
Network density		−0.60 (0.42)	−0.62 (0.42)	−0.65 (0.46)	−0.67 (0.47)
Network size		−0.30 (0.41)	−0.32 (0.41)	−0.23 (0.46)	−0.32 (0.46)
Gender (ref: Woman)	Man			0.23 (0.51)	0.22 (0.52)
	Non‐Binary			1.56 (0.63)*	1.55 (0.78)
Ethnicity (ref: White)	Asian			0.55 (0.57)	0.64 (0.57)
	Black			−0.15 (0.43)	−0.23 (0.43)
	Mixed			0.50 (0.41)	0.52 (0.41)
	Other			0.07 (0.33)	0.11 (0.33)
	PNTS			−0.26 (0.40)	−0.23 (0.41)
Student status (ref: Domestic)	International			0.92 (0.59)	0.86 (0.60)
	PNTS			−0.23 (0.33)	−0.20 (0.34)
*R* ^*2*^ *(residual SE)/adjusted R* ^*2*^		0.14 (3.09)/0.01	0.14 (3.08)/0.01	0.26 (3.06)/0.03	0.25 (3.09)/0.003
*F(df)*		1.06 (10, 68)	1.11 (10, 68)	1.11 (19, 59)	1.02 (19, 59)

*Note:* *p < 0.05. Showing coefficient (SE).

^a^ Gender heterogeneity was removed from the model.

^b^ Gender diversity was excluded from the model.

A final model considered how change in recalled social network attributes from pre‐university to university related to loneliness (Model 3a; Table 7). *Change* in social network attributes accounted for 25% of the variance in loneliness, *F*(11, 67) = 2.04, *p* = 0.038. Here, attributes associated with greater loneliness were reduced average perceived support and increased ethnicity heterogeneity, that is, participants' university network included more contacts of a different ethnicity to the participant than the pre‐university network. These associations remained significant after controlling for demographic characteristics, though the overall model no longer accounted for a significant proportion of variance in loneliness (Model 3b; Table 7). Loneliness was also associated with reduced relationship diversity, that is, participants were lonelier if the connections in their university network fell into a narrower range of relationship categories (e.g., friend, family, acquaintance) than connections recalled from the pre‐university network (Model 3a). This association did not remain significant when controlling for demographic factors (Model 3b).

**Table 7:** Findings from two regression models assessing the association between change in network characteristics over time and loneliness (Model 3), with (b) and without (a) controlling for demographics.

Parameter		Model 3a	Model 3b
Δ Gender EI		−0.17 (0.38)	−0.20 (0.40)
Δ Ethnicity EI		1.13 (0.39)**	1.26 (0.41)**
Δ Gender entropy		0.08 (0.39)	0.20 (0.47)
Δ Relationship type entropy		−0.80 (0.39)*	−0.76 (0.44)
Δ Average geographical distance		0.11 (0.37)	0.06 (0.44)
Δ Average support		−0.90 (0.42)*	−1.03 (0.48)*
Δ Average closeness		−0.42 (0.41)	−0.34 (0.46)
Δ % Online only relationships		−0.24 (0.37)	−0.19 (0.39)
Δ Relative offline/online interaction frequency		−0.04 (0.39)	−0.12 (0.41)
Δ Network density		0.26 (0.38)	0.39 (0.41)
Δ Network size		−0.70 (0.40)	−0.92 (0.43)*
Gender (ref: Woman)	Man		0.37 (0.45)
	Non‐Binary		0.77 (0.5)
Ethnicity (ref: White)	Asian		−0.26 (0.49)
	Black		−0.55 (0.40)
	Mixed		0.27 (0.40)
	Other		0.14 (0.32)
	PNTS		−0.32 (0.39)
Student status (ref: Domestic)	International		0.43 (0.59)
	PNTS		0.12 (0.30)
*R* ^*2*^ *(residual SE)/adjusted R* ^*2*^		0.25 (2.90)/0.13	0.34 (2.92)/0.11
*F(df)*		2.04 (11, 67)*	1.50 (20, 58)

*Note:* *p < 0.05, **p < 0.01. Showing coefficient (SE).

## 5 Discussion

This study quantified perceived social network churn during the transition to university. While young people experienced noticeable changes in their networks, these shifts were substantial but not extreme and occurred relatively consistently across demographic groups. Contrary to our hypothesis, network churn was not associated with loneliness. The composition of networks changed in several notable ways. Compared to their recalled networks before the transition, networks after the transition were larger, more geographically dispersed and included more peers from ethnic backgrounds that differed from their own. The post‐transition network was generally perceived as less emotionally close and less interconnected. Average support did not change. In line with our hypotheses, changes in two network characteristics were associated with loneliness: a decrease in perceived support provided by contacts and increased ethnic heterogeneity, indicating reduced ethnic homophily.

The transition to university appears to offer many young people a unique opportunity to diversify and expand their social world (Thomas et al. 2020). Our data confirms perceived increases in network size alongside increasing diversity of ethnicity and relationship type. Broader literature on social network development across the life course highlights both continuity and change: core social connections often remain stable, but peripheral networks are more fluid and responsive to life transitions (Antonucci et al. 2014; Wrzus et al. 2013). For example, retirement and relocation are typically associated with gradual reductions in network size and shifts towards kin‐based or geographically proximate relationships (Weiss et al. 2022), while parenthood can narrow networks and intensify contact with family (David‐Barrett et al. 2016). Other transitions, such as international migration or school‐to‐work transitions, are often marked by abrupt network disruption followed by rebuilding (De Schepper et al. 2023; Ryan et al. 2008). University entry shares the feature of sudden disruption but is distinctive in several ways: it typically occurs simultaneously for a large cohort of peers, within a highly concentrated social environment (e.g., halls, classes), and with structured opportunities (e.g., societies, sports clubs) that have the potential to actively facilitate network formation. This combination allows young people to rapidly expand and diversify their social networks in ways that are less characteristic of other abrupt life transitions (Priestley et al. 2025).

Past research has often framed network churn as a key risk factor for loneliness, particularly during major life transitions (Fischer and Offer 2020). We did not observe this association. This may reflect the developmental normalcy of churn at this life stage. Emerging adulthood often involves recalibrating social networks in response to shifting roles, routines, and settings. In this context, churn may be adaptive: it allows young people to shed unhelpful or mismatched connections and create space for new, more fitting relationships (Tulin et al. 2021). Network churn was considered in the whole and did not distinguish between weak versus strong ties or peripheral versus emotionally central relationships. Thus, high churn may not necessarily indicate meaningful social disruptions; there may have been substantial change in peripheral and weak ties, while strong, emotionally central ties were retained.

Compared to their pre‐transition networks, young people reported larger, more geographically dispersed networks that were less interconnected and felt less close, with greater diversity in relationship type and greater ethnicity heterogeneity. Networks were perceived by participants to have changed to include a greater proportion of acquaintances, compared to family or friends. This may be an artifact of recall bias, affecting the pre‐university network; participants may find it easier to recall weakly known acquaintances in their current network, than think back to acquaintances known months ago. However, the university network notably included a substantially smaller proportion of family; the increased proportion of acquaintances was almost exclusively replacing family and not friends. This pattern contrasts with other major life transitions, such as parenthood or retirement, which are typically associated with network contraction, increased homophily, and greater emotional closeness with a smaller core of connections (Wrzus et al. 2013). The university transition, by contrast involves a shift towards expansion and diversification, even at the expense of closeness and density. This may reflect the developmental salience of exploration in emerging adulthood; here forming weak connections and building heterogeneous networks can facilitate social integration and adaptation to new roles and settings (Arnett 2000, 2010).

These findings have implications for social convoy theory, which proposes that individuals maintain close‐knit groups of relationships that evolve through life transitions, providing emotional, practical, or informational support (Antonucci et al. 2014; Wrzus et al. 2013). While social convoy theory emphasizes stability in core relationships across transitions, our findings support transition induced network reconfiguration; during the transition to university, networks may undergo a period of marked expansion, diversification, and reduced closeness. This pattern may reflect a temporary loosening of the “convoy” as students recalibrate their social worlds, shedding prior peer connections and building new ones suited to their changing roles and environments. Importantly, although social convoy theory allows for peripheral changes alongside core stability, the breadth and heterogeneity observed here suggest that university entry may involve more profound restructuring than had been described in other transitions. Notably, perceived average support did not differ between pre‐transition and current networks, suggesting that despite turnover, a supportive core persists, in line with social convoy theory. Therefore, rather than signaling a breakdown of the convoy, these shifts may represent an adaptive reconfiguration, in which stable core relationships persist while the surrounding network temporarily expands to facilitate integration into new social contexts.

One of the clearest associations with loneliness was a perceived reduction in supportiveness within young people's social networks. Importantly, average support remained stable across the transition at a population level, suggesting that many students successfully maintain or rebuild supportive social ties despite substantial network turnover. However, students who perceived their social networks to have become less supportive also reported greater loneliness. This aligns with discrepancy models of loneliness, which emphasize the importance of relational quality rather than social contact quantity alone (Perlman and Peplau 1981; Qualter et al. 2015). While young people appeared able to tolerate considerable social network change, perceived reductions in support may represent an important marker associated with vulnerability during adaptation to university life. Universities may therefore benefit from interventions that not only facilitate social connection but also support students in building relationships characterized by emotional support, reciprocity, and meaningful connection.

Young people whose networks became more ethnically heterogeneous, that is, included a greater proportion of contacts from ethnic backgrounds different from their own, reported greater loneliness, even after adjusting for demographic variables such as ethnicity and student status (domestic vs. international). Homophily, that is, the extent to which an individual's social contacts share the same demographic characteristic as them, can foster belonging and shared identity (Jeroense et al. 2024; Krackhardt and Stern 1988). Thus, in predominantly white institutions, students from ethnic minority groups may experience heighten minority stress or feelings of social isolation (Crook 2025). While students value the opportunity to meet people from different backgrounds, they tend to look for people “like them” and find comfort in spending time with people who share their ethnicity and background (Priestley et al. 2025; Rakow et al. 2026, Under Review). Further research here is needed to test the stability of the association and understand the nature of the association.

### 5.1 Limitations

Several limitations should be considered when interpreting these findings. The sample size was modest (analytic *n* = 79), limiting statistical power for subgroup analyses. For instance, although the sample was ethnically diverse, it was not sufficiently large to robustly examine whether the association between changes in ethnic heterogeneity and loneliness differed across ethnic groups. The absence of associations across some models may reflect limited power. Network characteristics for the pre‐university period were reported retrospectively and may therefore be vulnerable to recall bias. Participants' recollections of earlier social relationships may have been reconstructed considering current experiences and emotional states, potentially affecting the accuracy of recalled network characteristics. Age was not included as a covariate due to missing data arising from a survey error. However, the sample was relatively age‐homogeneous, consisting predominantly of students aged 21 years and under, reducing the likelihood that age substantially confounded findings. While the study captured structural and compositional network change, it did not assess broader contextual factors (e.g., institutional climate, accommodation type, course structure) that may shape both network formation and loneliness.

The observational and correlational nature of the study precludes causal conclusions about the associations between perceived network change and loneliness. Although participants retrospectively reported network characteristics before and after entering university, neither network characteristics nor loneliness were experimentally manipulated. The observed associations may be bidirectional or may reflect unmeasured factors influencing both social networks and loneliness. For example, reduced perceived support may contribute to loneliness, but loneliness may also shape how individuals perceive or maintain their relationships. The findings should therefore be interpreted as associations rather than evidence that changes in network characteristics cause loneliness.

### 5.2 Policy Implications

These findings have implications for institutional policy and practice. Universities increasingly recognize student belonging and social integration as priorities within Whole University Approaches to mental health and wellbeing (Byrom et al. 2025; Hughes and Spanner 2024). Students perceived substantial network churn but churn itself did not predict loneliness. As such, messaging around the transition should normalize changing friendship networks and reassure students that social reorganization is expected during university adjustment. The transition to university expands and diversifies students' social worlds and universities may want to intentionally preserve and strengthen structured opportunities for network formation, supporting social integration as part of educational design. As supportive core relationships appear to remain important despite network restructuring, it may be beneficial to help students to maintain existing important relationships alongside developing new ones. Successful interventions do exactly this (Haslam et al. 2016). Reduced perceived support was associated with loneliness, indicating that support is needed that stretches beyond social contact. Efforts to strengthen relational quality, such as personal tutoring, peer support, mentoring schemes and small‐group educational practice, may be important. The association between increasing ethnic heterogeneity and loneliness further highlights that institutions must actively support social cohesion and belonging to ensure diverse student communities translate into inclusive student experiences (Aman et al. 2026; Armstrong‐McDowell et al. 2026). These findings reinforce the importance of institutional approaches that place social connection, belonging, and inclusive community‐building at the center of student wellbeing strategies.

## 6 Conclusion

Students perceived substantial network restructuring across the transition to university, but churn was unrelated to loneliness and average support remained stable. Greater loneliness was associated with perceived reductions in support and increased ethnic heterogeneity. Our findings highlight the complex and dynamic nature of young people's social networks during the transition to university. Our results underscore the nuanced role of diversity in students' networks.While exposure to difference can be enriching, it may also create challenges in establishing social connection, particularly when demographic shifts are mismatched with students' support needs or occur within institutions lacking sufficient mechanisms for cohesion. Taken together, these findings suggest that the university transition involves both the loss of old connections and the navigation of new, more diverse networks, requiring students to renegotiate how and with whom they maintain close bonds. Universities therefore have a critical role to play in supporting this process, not only by providing structured opportunities to meet others, but by actively fostering inclusive, integrated student communities that enable all students to feel connected, supported, and seen.

## Author Contributions

**Hannah Rachael Slack:** conceptualization, methodology, data curation, investigation, formal analysis, writing – original draft, writing – review and editing. **Nicola Byrom:** conceptualization, methodology, formal analysis, supervision, funding acquisition, project administration, writing – original draft, writing – review and editing. **Chloe Bate:** writing – original draft, writing – review and editing. **Aleksandra Hościłowicz:** investigation, writing–review and editing. **Sophie Homer:** writing – review and editing, funding acquisition. **Delia Fuhrmann:** conceptualization, methodology, writing – review and editing, funding acquisition. **Juliet Foster:** funding acquisition, writing – review and editing. **Sarah Crook:** funding acquisition, writing – review and editing. **Emily Long:** investigation, conceptualization, methodology, formal analysis, funding acquisition, writing – original draft, writing – review and editing, supervision, project administration.

## Conflicts of Interest

The authors declare no conflicts of interest.

## Supporting information


Supporting File

## Data Availability

The data that support the findings of this study are openly available in UK Data Service at https://reshare.ukdataservice.ac.uk/858287/.
