# Supplementary material for: From Familiar Faces to New Spaces: Perceived Social Network Change and Its Association With Loneliness During the Transition to University
Source: J Adolesc. 2026 Aug 6;98(7):2665–76. doi: 10.1002/jad.70249 (PMC13643633; doi:10.1002/jad.70249)
Supplement: Supplementary file 1 [file JAD-98-2665-s001.docx]

**Appendices**

**Appendix A. Missing data, outlier handling, and multicollinearity checks**

*Missing data*

To handle missing data, a listwise deletion approach was used. Of the recruited 90 participants, eleven participants were removed due to missing data across a combination of the loneliness, average distance and network density variables (see Appendix A1 for the percentage of missing data per variable). Twelve participants had missing age data due to a survey error. Hence, to preserve the analytical sample size, age was not included in the analysis. As the result of a survey error, three named contacts were falsely categorised as both lost and retained in the transition from the pre-university to the current university networks and hence, removed from the analyses.

*Outlier handling*

Outliers were identified using Tukey’s 1.5 IQR rule for Spearman’s rank correlation, paired samples t-tests, and Wilcoxon signed rank tests (Tukey, 1977). Outlier regression residuals were detected using the ±3 standardised residual rule (Fox, 2015) and influential residuals were identified using Cook’s distance. Removing outliers did not change direction or significance of the findings for the Spearman’s rank correlation, the paired samples t-tests and the Wilcoxon signed-rank tests. For all regression models, the findings remained stable after removing both outlier and influentials residuals.

*Multicollinearity checks*

Multicollinearity was assessed using Variance Inflation Factors (VIFs). The majority of VIFs were below the recommended threshold of 5, indicating that multicollinearity was not a major concern. Two VIFs were above this threshold, Gender heterogeneity and Gender diversity. See Appendices A2 and A3 for the VIF values.

Appendix A1. Missingness across all variables included in the analyses

| Parameter | Pre-university network (%) | Current network (%) |
| --- | --- | --- |
| Loneliness | - | 5.56 |
| Network churn | - | 0.00 |
| Gender heterogeneity (EI index) | 0.00 | 0.00 |
| Ethnicity heterogeneity (EI index) | 0.00 | 0.00 |
| Gender diversity (entropy) | 0.00 | 0.00 |
| Relationship type diversity (entropy) | 0.00 | 0.00 |
| Average geographical distance | 0.00 | 3.33 |
| Average support | 0.00 | 0.00 |
| Average closeness | 0.00 | 0.00 |
| % Online only relationships | 0.00 | 0.00 |
| Relative offline/online interaction frequency | 0.00 | 0.00 |
| Network density | 2.22 | 0.00 |
| Network size | 0.00 | 0.00 |
| Gender | - | 0.00 |
| Ethnicity | - | 0.00 |
| Student status | - | 0.00 |

Appendix A2. The Variance Inflation Factor (VIF) values for four regression models assessing the influence of pre-university (Model 1) and current network (Model 2) characteristics on loneliness, with (b) and without (a) controlling for demographics.

| Parameter | Model 1a | Model 1b | Model 2a | Model 2b |
| --- | --- | --- | --- | --- |
| Gender heterogeneity (EI index) | 2.68 | 3.40 | 5.77 | 9.99 |
| Ethnicity heterogeneity (EI index) | 1.24 | 1.82 | 1.42 | 2.42 |
| Gender diversity (entropy) | 2.78 | 3.23 | 6.38 | 7.66 |
| Relationship type diversity (entropy) | 1.88 | 2.31 | 1.73 | 2.21 |
| Average geographical distance | 1.14 | 1.45 | 1.47 | 1.97 |
| Average support | 1.71 | 2.32 | 1.53 | 1.78 |
| Average closeness | 1.82 | 2.03 | 1.79 | 2.28 |
| % Online only relationships | 1.40 | 1.53 | 1.50 | 1.88 |
| Relative offline/online interaction frequency | 1.21 | 1.44 | 1.77 | 2.18 |
| Network density | 1.56 | 1.70 | 1.47 | 1.79 |
| Network size | 1.83 | 1.95 | 1.44 | 1.78 |
| Gender |  | 1.25 |  | 1.45 |
| Ethnicity |  | 1.17 |  | 1.17 |
| Student status |  | 1.16 |  | 1.27 |

*Note.* VIF values are reported at the factor level for gender, ethnicity, and student status.

Appendix A3. The Variance Inflation Factor (VIF) values for two regression models assessing the influence of change in network characteristics over time (Model 3), with (b) and without (a) controlling for demographics.

| **Parameter** | **Model 3a** | **Model 3b** |
| --- | --- | --- |
| Δ Gender EI | 1.32 | 1.43 |
| Δ Ethnicity EI | 1.42 | 1.55 |
| Δ Gender entropy | 1.44 | 2.01 |
| Δ Relationship type entropy | 1.44 | 1.81 |
| Δ Average geographical distance | 1.29 | 1.80 |
| Δ Average support | 1.65 | 2.12 |
| Δ Average closeness | 1.53 | 1.91 |
| Δ % Online only relationships | 1.29 | 1.36 |
| Δ Relative offline/online interaction frequency | 1.38 | 1.55 |
| Δ Network density | 1.32 | 1.51 |
| Δ Network size | 1.47 | 1.70 |
| Gender |  | 1.11 |
| Ethnicity |  | 1.15 |
| Student status |  | 1.28 |

*Note.* VIF values are reported at the factor level for gender, ethnicity, and student status

Fox, J. (2015). *Applied regression analysis and generalized linear models*. Sage publications.

Tukey, J. W. (1977). *Exploring Data Analysis* (Vol. 2). Addison-Wesley.
